# Editorial: Chemistry and the circular economy

**DOI:** 10.3389/fchem.2023.1350994

**Published:** 2023-12-15

**Authors:** Paola Rizzarelli, Alessio Zuliani, Narendra Reddy

**Affiliations:** ^1^ Institute for Polymers, Composites and Biomaterials (IPCB), National Council of Research (CNR), Catania, Italy; ^2^ Institute for Chemical Research (IIQ), CSIC-University of Seville, Sevilla, Spain; ^3^ Center for Incubation Innovation Research and Consultancy, Jyothy Insitute of Technology Campus, Bengaluru, India

**Keywords:** circular ecomomy, bioproducts and biofuels, agricultural residues (ARs), clean processing, chemistry and industry

Chemistry stands as the cornerstone for life’s sustenance and progress on our planet, offering solutions to humanity’s challenges throughout history. In the modern era, Chemistry is an integral part of daily life, influencing science and technology. While its foundations lie in natural elements, synthetic chemistry advancements have excelled in cost, manufacturing ease, scalability, and consistency. Yet, concerns about health and environmental effects urge a reevaluation of the exploitation and applications of natural sources. Since 2000, global concerns like climate change and resource depletion have fueled extensive exploration for alternative solutions to using natural resources, especially amid environmental disasters, pandemics, and geopolitical struggles. One of the major approaches being considered to meet and overcome these challenges is by maximizing the use of natural resources and minimizing the generation of waste (Tseng et al., 2020). In this context, the concept of circular economy, where the waste and coproducts generated by one process are considered as inputs for another one and converted into value added products through clean and green approaches, is being aggressively pursued and adopted (Velenturf and Purnell, 2021).

Based on current statistics, the global agriculture market is expected to cross US$ 5 trillion by 2028. Consequently, the generation of byproducts and coproducts during agricultural production and processing is also set to increase steeply (Kircher, 2019; Díaz-Bonilla, 2023). Stems, leaves and husks generated during agricultural production and several byproducts, including proteins and carbohydrates, supplied as coproducts during agricultural processing are available in large quantities at low cost. These byproducts and coproducts have the essential constituents to be converted into monomers, polymers, compounds and further into any desired substance through specific chemical transformations, ranging from carbonyl reductions to carboxyl decarboxylations, hydroxyl oxidations, glycosylation, and transglycosylation (Li et al., 2014; Donner et al., 2021). For instance, corn-based biorefineries utilizing starch, corn husks, corn cobs, distillers dried grains and other coproducts have generated biofuels (ethanol, methane), biopolymers (polylactide), amino acids, animal feed and enzymes. Similarly, sugarcane based biorefineries have excelled in the production of biofuels, paper, fertilizers, animal feed, chemicals such as acetic acid, cosmetics and perfumes (Gerrior et al., 2022). Not only regular food crops but also non-food products such as poultry feathers have been considered for production of composite, plastics, biofuels, etc. Based on these approaches, it is evident that there is unlimited scope for extending the biorefinery and circular economy concepts to almost all agricultural byproducts and coproducts.

To achieve successful utilization and exploitation of renewable resources, the development of innovative and efficient conversion processes is essential. The purpose of the present Research Topic, “Chemistry and The Circular Economy”, aligns with this goal. It displays the continuous progress and the potential of chemistry in addressing global challenges by bringing together research articles that explore advancements in energy storage, hydrogen production, polymer synthesis, and biochar utilization.

In the realm of energy storage, researchers have dedicated over 2 decades to refining biomass through rapid pyrolysis technology. However, challenges emerge as the primary product, biomass pyrolysis oil, grapples with low energy density and poor thermal stability. The intrigue deepens as exploration leads towards catalytic reforming as a transformative avenue, offering a potential solution for the subsequent utilization of bio-oil in hydrogen production. In a captivating mini review, Zhang focuses on the innovative technique of CO_2_ adsorption-enhanced catalytic reforming, presenting itself as a beacon of hope for efficient and sustainable hydrogen generation. The review briefly introduces recent progress in biomass catalytic reforming hydrogen production technology, covering mechanisms, catalyst selection, new processes, and technology developments. It also addresses current challenges in this field, providing potential ideas and directions for future development.

Shifting the focus to the captivating world of bio-derived polymers, the synthesis of poly-γ-glutamic acid (γ-PGA) comes into play. This water-soluble marvel holds promise as a game-changer in various applications. Its potential spans from the realms of drug delivery and cosmetics to bioremediation and wastewater treatment, weaving a tale of sustainability and versatility. Parati et al. explores the synthesis of γ-PGA from scalable macroalgal biomass assessing the influence of pre-treatment type, macroalgal species, and collection time on brown seaweed cultivated under controlled conditions in Scotland. *Laminaria digitata*, *Saccharina latissima*, and *Alaria esculenta*, three brown seaweed species native to the United Kingdom, are analyzed for their variation in carbon, antioxidant, protein, and ash content. The research evaluated the effect of different algal species on the yields and chemical composition of γ-PGA produced by *Bacillus subtilis natto*, also investigating the variations during the specific cultivation period.

Li et al. present a review delving into the promising realm of biochar, an environmentally friendly material drawing attention for its potential use as a potassium ion anode in batteries. The manuscript explores the transformative possibilities of biochar through modifications, particularly highlighting atomic doping to enhance electrochemical performance. This type of materials exhibits not only efficient energy conduction but also increased potassium storage capacity. The review covers the application of atomically doped biomass carbon materials (BCMs) and recent advancements in pure BCMs, emphasizing considerations for simpler production, reduced costs, and environmental protection. Key challenges in developing BCMs, such as irreversible potassium buildup and electrolyte breakdown, are discussed and potential solutions have also been proposed.

Supporting the circular economy model, a beacon of hope for combating the environmental impact of poly(ethylene terephthalate) (PET), a commonly produced and consumed polymer, comes from (Gabrielli et al.). Their proposed approach involves a microwave-assisted recycling process, focused on aminolysis of PET waste to create polyurethane acrylate (PUA) derived coatings. The process utilizes substituted β-hydroxy amines for chemical depolymerization of PET waste, employing a sodium acetate catalyst. The first step achieves efficient depolymerization of PET within a short time, resulting in the formation of terephthalimide diols. This environmentally friendly method enables the synthesis of a diverse range of diol monomers with customizable organic structures suitable for PUA UV-curable coatings.

In this tapestry of research, each article of the present Research Topic not only unravels scientific complexities but also paints a vivid picture of innovation, sustainability, and the transformative power of human ingenuity in the pursuit of a brighter, greener future.
